# Tackling HIV by empowering adolescent girls and young women: a multisectoral, government led campaign in South Africa

**DOI:** 10.1136/bmj.k4585

**Published:** 2018-12-07

**Authors:** Hasina Subedar, Sarah Barnett, Tsakani Chaka, Sibongile Dladla, Ellen Hagerman, Sarah Jenkins, Gertrude Matshimane, Kerry Mangold, Busi Msimanga, Ruth Pooe, Lebogang Schultz, Yogan Pillay

**Affiliations:** 1 National Department of Health, Pretoria, South Africa; 2 SB Consultancy World, Bristol, UK; 3 Office of the Deputy President, The Presidency, Pretoria, South Africa; 4 US Centre for Disease Control and Prevention, Pretoria, South Africa; 5 Equality Networx, Johannesburg, South Africa; 6 Clinton Health Access Initiative, Pretoria, South Africa; 7 South African National AIDS Council, Pretoria, South Africa; 8 World Health Organization, Pretoria, South Africa; 9 Department of Social Development, Pretoria, South Africa; 10 United Nations Population Fund, South Africa Country Office Pretoria, South Africa

## Abstract

**Hasina Subedar and colleagues** describe the intersectoral collaboration enabling She Conquers, a three year national campaign rolled out across South Africa, to tackle the multiple drivers of the high rates of HIV infection among adolescent girls and young women

Despite a recent fall in new infections, South Africa still has the largest HIV epidemic in the world and has not achieved the 50% reduction envisaged in its national strategic plan for 2012-16.^1^ ^2^ Adolescent girls and young women are disproportionally affected by HIV, with prevalence among 20-24 year olds three times higher in women (16%) than in men (5%), and females aged 15-24 years accounting for 37% of new infections.^1^ ^3^ Amid the competing priorities for HIV funding, the current national plan (2017-22)^2^ calls for urgent focus on adolescent girls and young women.

Although many organisations and government departments target adolescent girls and young women, action has often been piecemeal, resulting in duplication of effort, funds not allocated strategically, and limited impact. On World AIDS Day 2015, South Africa’s deputy president called for a collective and collaborative response to the high rates of HIV and its key drivers among adolescent girls and young women.^4^ In June 2016, the government launched the three year She Conquers campaign.^5^ The campaign seeks to reduce HIV infections, improve overall health outcomes, and expand opportunities for adolescent girls and young women to decide their own futures (table 1). The campaign moves beyond a focus on disease transmission and associated stigma to a narrative of power (see suppl 1 on bmj.com).

**Table 1:** Aim, objectives, and targets of She Conquers campaign

Aim	Objectives	Targets to be achieved over three years (2016 to 2019):
To reduce HIV infections, improve overall health outcomes, and expand opportunities for adolescent girls and young women to decide their own futures	To reduce new HIV infections among adolescent girls and young women aged 15-24 years	To decrease HIV infections by at least 30%: from 90 000 a year to less than 60 000 a year
	To reduce the incidence of teenage pregnancy (under 18s)	To decrease births to under 18s by at least 30%: from 73 000 a year (2015) to 50 000 a year
	To increase retention of girls in school until completion of grade 12	To increase school retention by 20% (baseline of 4% dropout in 2010)
	To reduce sexual and gender based violence experienced by adolescent girls and young women	To decrease sexual and gender based violence by 10% (2012 baseline: 7.7% for age 15-19; 7.3% for age 20-24)
	To increase economic empowerment of adolescent girls and young women	To increase youth employment by 10% (baseline 36.9% in 2015)

She Conquers primarily targets women aged 15-24 years, although interventions also target others in the HIV transmission cycle, such as older men and women.^6^ Core interventions are implemented by a diverse group of partners to collectively tackle the social and structural determinants of HIV, and include programmes on sexual and reproductive health, HIV testing, gender based violence, positive parenting, and post-schooling education and employment (fig 1). South Africa’s current deputy president provides high level leadership for the campaign.

**Fig 1:**
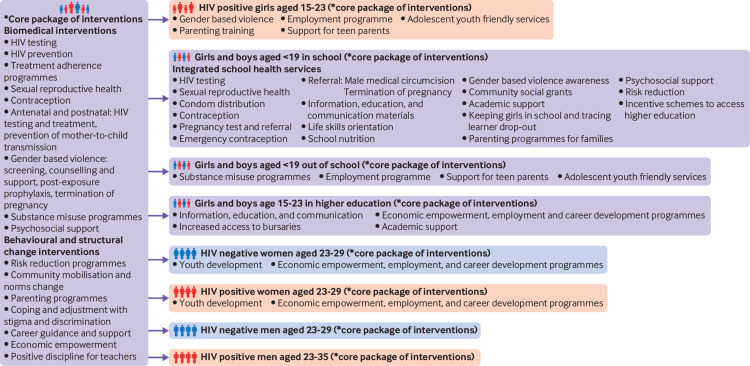
She Conquers core package of interventions

This case study explores the nature of the intersectoral collaboration within She Conquers, highlighting the success factors, limitations, and challenges as well as the lessons learnt. The insights we report may be relevant not only for future strengthening of the campaign but also for others seeking to collaborate across sectors to tackle health and development challenges. Methods for the case study analysis were informed by a guide developed by the Partnership for Maternal, Neonatal, and Child Health^7^ and included a review of literature, as well as one-to-one in-depth interviews with key stakeholders. Details of our methods are given in supplement 2 on bmj.com. A multistakeholder review meeting was held to validate the content of the case study.

## Key achievements of She Conquers

All three levels of government (national, provincial, and district) have engaged with the campaign, and She Conquers has managed to motivate government departments as well as a diverse mix of stakeholders from civil society, development organisations, private sector, and academic institutions to align. The campaign is being rolled out across all nine provinces in South Africa in three phases. It is currently in phase one, which includes the 22 subdistricts with the highest HIV burdens, with phase two due to expand to 31 additional prioritised subdistricts, and phase three to include remaining subdistricts in order of priority. Box 1 lists what has been achieved so far.

Box 1 **Progress under She Conquers**

- Over 20 government departments and 100 partner organisations have agreed to align under She Conquers
- Programmes for adolescent girls and young women account for over three billion South African rand ($200m; £160bn; €180bn)
- She Conquers covers a total of three million young women, within 22 priority subdistricts, across all nine provinces

**Progress on She Conquers interventions (1 July 2016-31 December 2017) 8**

- More than 700 000 adolescent girls and young women have had an HIV test
- 26 000 adolescent girls and young women who tested HIV positive were linked to care
- Over 560 000 adolescent girls received life skills and sexual education
- More than 90 000 adolescent girls and young women received post-violence care
- Nearly 19 000 young boys and girls participated in violence prevention programmes
- More than 72 000 adolescent girls received support to remain in school
- More than 19 000 adolescent girls and young women attended economic strengthening programmes
- Over 6000 completed a parenting programme (including teen parents)

## How multisectoral collaboration was achieved

We identified six factors that may have been important in ensuring successful alignment: strong strategic planning; committed high level leadership; alignment to existing coordinating structures; leveraged resources; mobilisation of partners for integration; and engagement with adolescent girls and young women to ensure a relevant and responsive campaign.

### Strong strategic planning

Given the number of stakeholders, resources involved, wide geographical coverage, and that programmes were not structured to promote collaboration, strong strategic planning was essential from the outset to promote alignment and foster partnerships. High HIV rates among adolescent girls and young women are principally linked to social determinants, including poverty, unemployment, gender inequality, and alcohol and substance misuse (suppl 3 on bmj.com). A large scale phylogenetic study from South Africa revealed the cycle of HIV transmission among young women (box 2).^6^ This evidence was presented at a meeting of senior leaders in the She Conquers campaign before formal peer review publication to inform the development of the campaign, including the campaign strategy, objectives, theory of change, and core package of interventions.

Box 2 **Key findings from a community phylogenetic study of HIV transmission**

A phylogenetic mapping of the HIV transmissions pathway conducted in Hlabisa, KwaZulu-Natal in 2014-15 provided an explanation for the high incidence and prevalence among adolescent girls and young women aged 15 to 24.^6^ This age group tend to engage in sexual relationships with men roughly eight years older than themselves; the men have higher prevalence levels and are therefore more likely to transmit HIV to their younger partners. In their 20s, young women who have already been exposed to HIV from previous older partners then often have sexual relationships with men in their same age group, thereby continuing the cycle of infection.

**Fig 2:**
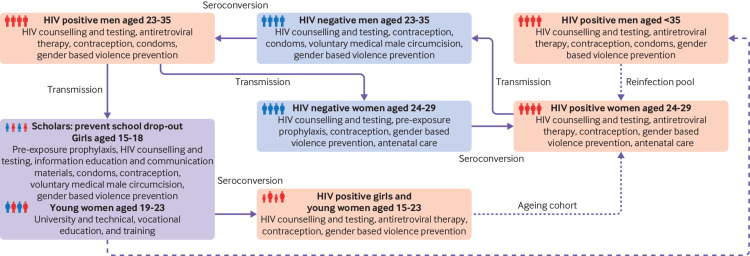
Action to break the cycle of HIV transmission among young women

The issues to be tackled included teenage pregnancy, gender based violence, gender equality, keeping girls in school, and women’s socioeconomic empowerment (fig 2). A stakeholder mapping exercise during the planning phase identified areas that overlapped or complemented, and the campaign was designed to build on existing programmes. The package of interventions (fig 1) identifies actions to be taken by stakeholders, while allowing for adaptation in targeting specific groups and geographical areas. The core package of interventions is complemented by additional materials, including a monitoring and evaluation framework, roadmaps to services, communications material, and website. All campaign programmes were aligned under a common name and logo, using consistent branding to achieve a unified message.

As part of the strategic planning process, the campaign was aligned with national strategies, including the National Youth Policy 2015-20.^9^ The campaign objectives were embedded within the national strategic plan, which articulates South Africa’s strategy for encouraging all levels and sectors of society to tackle the HIV epidemic. The plan connects She Conquers to broader national policies that drive the overall vision for fostering collective actions to transform society, including the mid-term strategic framework and the national development plan. 

To maximise promotion of the issues relevant to She Conquers, many of its campaign activities are aligned with existing campaigns, such as Youth Day in June, National Women’s Day in August, and World AIDS Day in December. Phased roll-out also provides an opportunity for others to learn from the best practices of phase one districts.

### Committed high level leadership

President Cyril Ramaphosa, who was South Africa’s deputy president when She Conquers started, has been a key spokesperson and figurehead for the campaign, bringing political commitment from the highest level. This proved vital for collaboration as leadership was not assigned to just one sector. When he was inaugurated as president in 2018 he stressed the importance of the She Conquers campaign in his State of the Nation speech,^10^ further raising its profile. High level publicity resulted in widespread commitment to She Conquers from the outset, with strong representation by development partners, donor agencies, government ministers, and departments at the launch.

The high level leadership stimulated a sense of responsibility, political buy-in, and collective commitment from diverse stakeholders working on programmes for adolescent girls and young women. Given the competing priorities for HIV funding, it maintained the focus on young women. In 2016, President Zuma instructed every government department to ensure their programmes target young people, and consensus is growing among leaders at all levels and across the political spectrum about the importance of addressing the challenges faced by young people.^11^

### Aligned to existing coordinating structures

**Fig 3:**
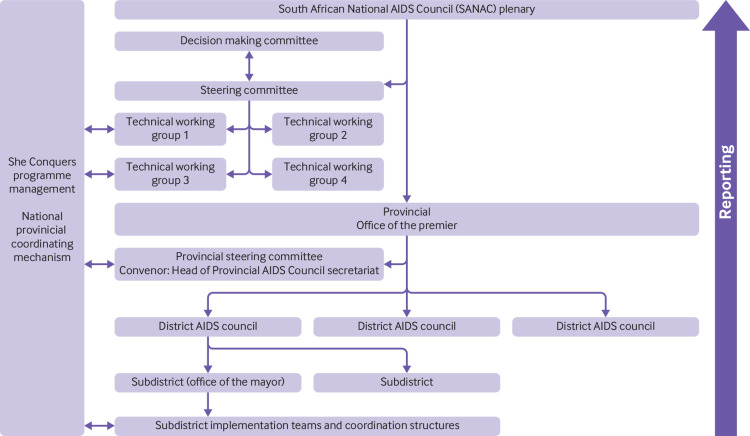
She Conquers coordinating structure, aligned to the South African National AIDS Council (SANAC) coordinating structure

The campaign is built on existing coordinating structures and mechanisms that drive the country’s efforts to tackle HIV (fig 3). These structures already bring together government, civil society, and the private sector, and cascade from national to subdistrict level. At operational level, the South African National AIDS Council (SANAC) coordinates both the national HIV response and She Conquers. The SANAC inter-ministerial committee, chaired by the country’s deputy president, provides political oversight for tackling HIV and She Conquers, leading discussions to review progress, identifying and overcoming challenges, and encouraging government departments to align with She Conquers to facilitate engagement.

At subnational levels, the provincial and district AIDS councils have a lead role in coordinating programmes working with young people to foster a targeted response, and within each province the campaign is led by the premier’s office. At the start of the campaign, provincial councils consulted potential partners, including representatives of civil society, youth organisations, government departments, and implementing partners. Discussions focused on identifying priority subdistricts and existing coordinating structures that could be drawn on for the campaign.

New coordination structures have been established to support better alignment and to delineate roles and responsibilities. These include a national steering committee, a national decision making committee, provincial steering committees, and subdistrict implementation teams. Additional subcommittees on monitoring and evaluation, communications, and innovation existed during the planning phase to devise strategies and develop materials. The committees bring together stakeholders and allow them to develop strategy collectively. They provide an in-depth understanding of what other stakeholders and partners are doing, enabling the forging of new relationships and thus expanding collaborations around adolescent health issues.

### Leveraged resources

Substantial investment in programmes for adolescent girls and young women existed before the campaign: in 2015, a one-off resource mapping exercise revealed over three billion rand was invested across various sectors. This derived largely from three major donors (the Global Fund, the US President’s Emergency Plan for AIDS Relief (PEPFAR), and KfW Development Bank. No dedicated campaign funding existed, however, so strategic planning was necessary to ensure that existing investment would help the campaign reach its objectives. 

Partners agreed that coming together under the campaign to coordinate and leverage existing financial and human resources would reduce duplication of efforts and produce better value for money. Partners would take responsibility for specific aspects of the campaign’s launch and implementation to enable the development of materials that aid collaboration and raise the campaign’s profile (such as logos, website, branding guide, promotional materials, stakeholder mapping, roadmap, communications strategy, and monitoring and evaluation frameworks).

### Aligning partners

Partners acknowledged that before the campaign they were working in silos, competing for resources, and failing to appreciate the benefits of collaboration. The campaign’s ability to mobilise over 120 government departments and partners to act together is a crucial achievement. The integration of large scale programmes, such as Global Fund and PEPFAR, was essential since they were already operating in priority subdistricts. This was partly achieved through strong advocacy: the need to focus on adolescent girls and young women, and to do so collaboratively, was repeatedly emphasised by the deputy president and the inter-ministerial committee. The campaign is further expanding its reach because of encouragement by donors.

Headlines from a 2013 survey showing that every week 2363 women aged 15-24 become infected with HIV in South Africa,^12^ which compared very unfavourably with other African countries, increased the willingness of development partners to collaborate. Further evidence raised awareness of the effects of new infections beyond health: on the economy, the job market, and the wellbeing of society.^3^ Partners recognised that joint benefits could accrue by aligning their programmes and broadening the reach and depth of interventions to tackle issues affecting adolescent girls and young women across multiple sectors.

The fact that government departments are becoming more sensitised to issues facing adolescent girls and young women and the need to work collaboratively is encouraging since working through existing structures improves sustainability. Additional key motivators for partners to align include increased public profile, opportunities for networking and joint collaboration, and access to donor resources being restricted to groups aligning with national strategies. However, the level of collective engagement varies, often because of geographical and political dynamics. Engagement can be encouraged by the appointment of a focal person to facilitate coordination and collaboration among partners within the district or province.

### Engaged adolescent girls and young women 

Young people have been engaged in She Conquers from the outset. They were involved in branding for the campaign, ensuring the name and logo were youth friendly (box 3). In the campaign’s first year, youth consultations were held across all nine provinces through the offices of the premier, enabling the specific concerns of young people to be identified in each province. Context affects how women and girls experience the campaign (supplement 3), and it is important that the campaign is flexible enough to allow local adaptations. Regular youth engagement occurs at the local level, where She Conquers partners assume responsibility.

Box 3 **Examples of youth engagement within She Conquers**

*Campaign logo—*Young people participated in a two day workshop to develop a logo that resonated with them. Four participants were nominated to work with the graphic designer, and a final version was shared with everyone who attended the workshop for approval
*Campaign name—*The campaign was launched with the logo, but without a name. At the launch, a competition for the name was announced by the deputy president and flyers were distributed with the details. A group of young people identified shortlisting criteria and shortlisted the final four campaign names
*Campaign launch—*Thousands of young people attended the launch from all over the country
*Communications—*During the first year of the campaign the lack of unified messages around the five objectives was identified as a gap. Flow Communications, in collaboration with the United Nations Population Fund (UNFPA), established a brand council to develop messages. The council includes people in the target groups who have not had previous exposure to health and behaviour change communication work
*Social media—*A youth led process on social media developed campaign messaging to engage other young people. During July 2018, the campaign was trending second only to the World Cup
*Peer to peer—*Johnson & Johnson, in collaboration with UNFPA, launched the DREAMS Thina Abantu Abasha programme (Zulu for “we the youth”), a youth led, peer-to-peer initiative aimed at empowering young people to reduce the rate of new HIV infections in KwaZulu-Natal and Gauteng through various interventions. It is based on the premise that no action of empowering young people should take place without their direct involvement

## Limitations and challenges

Several challenges have been experienced during the first 18 months of the campaign. Political and funding problems meant that some implementing partners were unable to offer the full package of sexual and reproductive health services in all districts. Although a systematic approach to tracking progress around campaign objectives was planned to strengthen stakeholder alignment, this has been challenging because each partner and government department has its own reporting requirements and timelines, and the lack of dedicated core funds has hampered the development of an integrated national reporting mechanism. Not all government departments have fully engaged, resulting in a lack of coordinated action on some key issues, such as gender based violence.

Furthermore, even though the programme had high level buy-in from government departments, strong leadership at provincial and district levels was less consistent. Some provinces have key staff who are motivated to systematically push the She Conquers agenda as part of their work, but commitment varies and it is not always possible to engage reliable local staff or to integrate the campaign into existing coordinating structures. The priority given to the campaign, and the speed of roll-out, has therefore varied between provinces and districts.

One of the biggest challenges facing the campaign is the lack of dedicated resources for sustained youth engagement at all levels. Engagement is hampered by the shortage of strong youth networks and the lack of a common platform for young people. Although the AIDS councils offer platforms at the provincial and district level, some do not function or do not engage young people. Concerns have been raised that the campaign primarily engages with youth from cities and fails to represent diversity, including those with lower levels of education and vulnerable groups. In April 2018, the adolescent and youth HIV prevention summit acknowledged the need to strengthen youth participation in the campaign, including drawing more on existing youth engagement programmes run by civil society or development partners. Although some civil society organisations convene youth discussions, stronger coordination of youth led action under She Conquers is required.

Lastly, although the campaign’s primary target is girls and young women aged 15-24 years, phylogenetic work confirmed that older men and women also need to be included.^6^ The core package of interventions also targets males, but concerns have been raised that the focus on adolescent girls and young women is excessive and that male behaviour needs more attention—for example, in relation to gender based violence and condom use, and their connection to patriarchy. This has led some to question the appropriateness of including the feminine pronoun “She” in the campaign’s name.

## Lessons learnt

*Leadership—*Ongoing leadership from the deputy president and engagement by senior department leaders promoted widespread engagement in the campaign at all levels. This was essential for multisectoral collaboration within government. In addition, champions were needed at all levels of government to convince all participants of their ability to take action and to promote a collaborative attitude and a shared vision.

*Strategic planning—*Strong national strategic planning was required from the outset to manage the large number of programmes targeting adolescent girls and young women, especially as they had not been designed to align to one another and have different timeframes and reporting systems. Effective implementation of the strategy required clear demarcation of roles and responsibilities, as well as accountability and coordination structures at the national, provincial, district, and community levels.

*Pooled resources—*With a lack of dedicated campaign resources, the campaign needed to effectively use the extensive resources already allocated to young women and assessed how their use could be optimised by identifying key stakeholders, their activities, and their contributions at national, provincial, and district levels.

*Learning from positive examples—*The effectiveness and reach of the campaign have differed among provinces and districts. The campaign tries to draw on the experiences and achievements of stronger districts to support less successful areas. This includes sharing materials and information, reporting best practices and lessons at meetings, and identifying people to drive particular elements of the campaign.

*Youth engagement—*Although the scale of the campaign prevents it from being youth led, the importance of youth participation has always been acknowledged. It is difficult to develop messaging that appeals to all young people, but the campaign takes into account their heterogeneous nature and finds innovative ways to hear the voices of marginalised groups, to ensure that the campaign can achieve the widest possible effect.

## Future directions 

There is a strong expectation that existing partners will continue to invest money and human resources, and that new partners will agree to align under the campaign, ensuring its sustainability. Discussions are under way about establishing a formal national coordination structure for the campaign to ensure that goals and objectives are achieved. The lack of an integrated reporting system has hampered tracking progress towards objectives, and the campaign intends to leverage resources for this, as well as for stronger youth engagement. The campaign plans to build on existing youth partnerships through civil society and to provide more support to enable youth to advocate as a collective. Although the term “campaign” suggests a limited and time bound effort, the project goals require and deserve a longer term footing and even wider application.

**Key messages**

- The She Conquers campaign has used extensive collaboration across sectors to tackle the social and structural determinants of HIV among girls and young women in South Africa
- Extensive advocacy, including high level leadership, helped to mobilise support for the campaign
- Activities were coordinated through existing national, provincial, district, and subdistrict structures
- She Conquers exploited existing resources to deliver key programme goals
- Partners’ programmes were aligned with national policies, campaign objectives, campaign theory of change, and a core package of evidence based interventions
- Communities were mobilised using common messaging, facilitating youth involvement and participation

## Web Extra

Extra material supplied by the author

Supplement 1: Illustration of theory of change for the campaign

Supplement 2: Methods for the case study

Supplement 3: Key challenges facing young women and adolescent girls

## Notes

See www.bmj.com/multisectoral-collaboration for other articles in the series.

## Notes

Contributors and sources:This article is based on finding from a review of the mulitsectoral collaborative work of She Conquers, commissioned by PMNCH and to which all authors contributed. HS, SB, and EH developed this case study based on the information collected, with inputs from YP, TC, SD, SJ, GM, KM, BM, RP, and LS. All authors undertook critical revisions of the manuscript drafts. HS is responsible for the overall content as guarantor.

## References

[1] Avert. Global information and education on HIV and AIDS. https://www.avert.org/professionals/hiv-around-world/sub-saharan-africa/south-africa

[2] *The national strategic plan for HIV, TB and STIs 2012-2016*. NDP, 2030. 2018.

[3] Shisana O Rehle T Simbayi LC South African national HIV prevalence, incidence and behaviour survey, 2012. HSRC Press, 2014. 10.2989/16085906.2016.1153491 27002359

[4] Key note address by Deputy President Cyril Ramaphosa during the commemoration of World AIDS Day, 1 December 2015. https://www.gov.za/speeches/deputy-president-cyril-ramaphosa-world-aids-day-2015-commemoration-1-dec-2015-0000

[5] She Conquers. http://sheconquerssa.co.za

[6] de Oliveira T Kharsany AB Gräf T Transmission networks and risk of HIV infection in KwaZulu-Natal, South Africa: a community-wide phylogenetic study. Lancet HIV 2017;4:e41-50. 10.1016/S2352-3018(16)30186-2 27914874 PMC5479933

[7] PMNCH. Methods guide for country case studies on successful collaboration across sectors for health and sustainable development. 2018. http://www.who.int/pmnch/knowledge/case-study-methods-guide.pdf

[8] She Conquers Campaign Joining the dots progress report 2018.

[9] The Presidency, Republic of South Africa. National youth policy 2015-2020. http://www.thepresidency.gov.za/download/file/fid/58

[10] Reply by President Cyril Ramaphosa at the state of the nation address, 20 Feb 2018. http://www.thepresidency.gov.za/speeches

[11] Key note address by President Jacob Zuma on the Presidency budget vote debate, National Assembly, Cape Town 4 May 2016. http://www.thepresidency.gov.za/speeches/address-president-jacob-zuma-occasion-presidency-budget-vote-debate%2C-national-assembly%2C-0

[12] UNAIDS. Preventing HIV in adolescent girls and young women. Guidance for PEPFAR country teams on the DREAMS partnership. 2015. http://ghpro.dexisonline.com/sites/default/files/PEPFAR%20Final%20DREAMS%20Guidance%202015.pdf

