# Supplementary figures and images for: Tackling HIV by empowering adolescent girls and young women: a multisectoral, government led campaign in South Africa
Source: BMJ. 2018 Dec 7;363:k4585. doi: 10.1136/bmj.k4585 (PMC6284473; doi:10.1136/bmj.k4585)

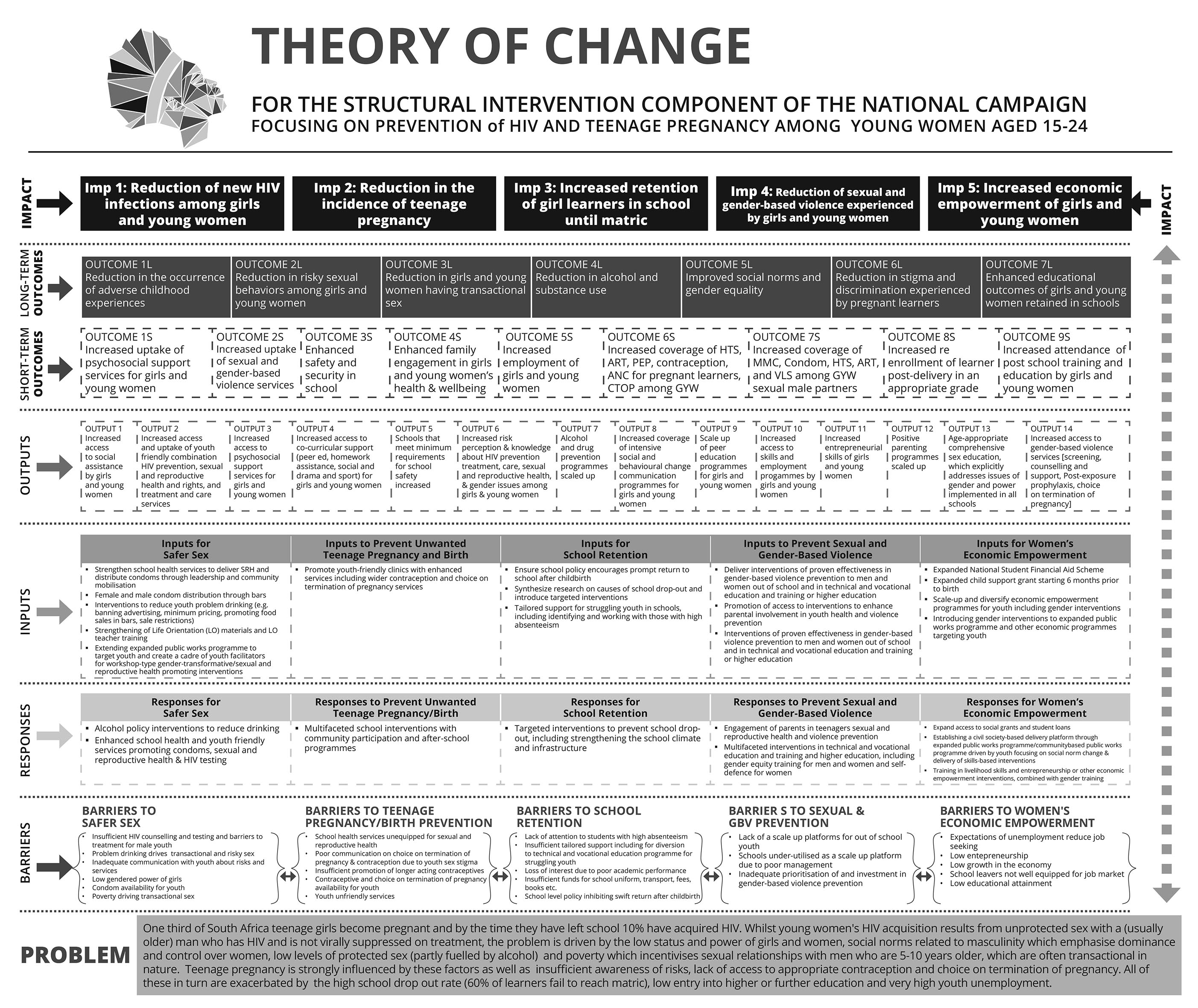

Supplement: Supplementary file 1 — Supplement 1: Illustration of theory of change for the campaign [file subh047331.ww1.jpg]
