# Supplementary material for: Tackling HIV by empowering adolescent girls and young women: a multisectoral, government led campaign in South Africa
Source: BMJ. 2018 Dec 7;363:k4585. doi: 10.1136/bmj.k4585 (PMC6284473; doi:10.1136/bmj.k4585)
Supplement: Supplementary file 2 — Supplement 2: Methods for the case study [file subh047331.ww2.pdf]

Supplement 2. Methods for developing the case study

The development of this case study followed the standard methodological approach outlined in a case study methods guide.[1] The process included three phases: 1) document review; 2) key informant interviews; and 3) a multistakeholder review. A semi-structured questionnaire in the case study methods guide provided the structure for collecting, organizing, and analysing the data, undertaken between May and July 2018.

1) Document review: A review of relevant programme reports, evaluations, and other peer-reviewed and grey literature was undertaken in May and June 2018, identifying relevant information, as per the semi-structured questionnaire in the case study methods guide.
2) Key informant interviews: A national consultant conducted key informant interviews with stakeholders who were involved with the She Conquers campaign, including representatives from government (Presidency, Ministry of Health, the South African National Aids Council), civil society, donors, academic institutions, and the private sector. Twenty interviews were conducted using an interview guide structured around the semi-structured questionnaire in the case study methods guide.
3) Multistakeholder meeting: In July 2018, a small multistakeholder meeting was held in Pretoria. Eight stakeholders attended from the National Departments of Health and Social Development, as well as development partners, and donors. The multistakeholder review process drew on both the methods used in the first Success Factors study series[2] and the PMNCH guide for multistakeholder dialogues.[3] The purpose of the multistakeholder meeting was to review and discuss the findings from the first draft of the case study and to make recommendations for edits and changes.

Limitations
The She Conquers campaign engages are large number of stakeholders. While the key informant interviews and multistakeholders engaged a wide range of stakeholders across sectors, it was not feasible to include all partners in the case study process. Although this may have been to the exclusion of diverse opinions, the methods did enable the triangulation of information and perspectives and was representative of a collaborative exercise.

References

1. PMNCH. Methods guide for country case studies on successful collaboration across sectors for health and sustainable development. 2018.

http://www.who.int/pmnch/knowledge/case-study-methods-guide.pdf
2. Frost L, Hinton R, Pratt BA, et al. Using multistakeholder dialogues to assess policies, programmes and progress for women's, children's and adolescents' health. *Bull World Health Organ* 2016;94(5):393-5. doi: 10.2471/BLT.16.171710 [published Online First: 2016/05/06]
3. PMNCH and WHO. Multistakeholder dialogues for women's and children's health: a guide for conveners and facilitators. 2014. http://www.who.int/pmnch/knowledge/publications/msd_guide.pdf
