# Supplementary material for: Tackling HIV by empowering adolescent girls and young women: a multisectoral, government led campaign in South Africa
Source: BMJ. 2018 Dec 7;363:k4585. doi: 10.1136/bmj.k4585 (PMC6284473; doi:10.1136/bmj.k4585)
Supplement: Supplementary file 3 — Supplement 3: Key challenges facing young women and adolescent girls [file subh047331.ww3.pdf]

Supplement 3. Key challenges facing adolescent girls and young women in South Africa

The table below highlights the context and drivers of, the five key objectives of She Conquers: HIV, teenage pregnancy, school retention, sexual and gender-based violence, and youth unemployment.

**HIV**	***Context:***<br>▪ South Africa has the most severe HIV epidemic in the world: 19% of the global number of people living with HIV, 15% of new infections, and 11% of AIDS-related deaths[1]<br>▪ The total number of persons living with HIV in South Africa increased from 4.94 million in 2002 to 7.06 million in 2017[2]<br>▪ Men who have sex with men, transgender women, sex workers, and people who inject drugs have the highest HIV prevalence rates (e.g. 58% of sex workers are HIV positive)[1]<br>▪ Approximately one-fifth of South African women of reproductive age (15–49 years) are HIV positive[2]<br>▪ Adolescent girls and young women aged 15–24 accounted for 37% of new infections in 2016[1]<br>▪ HIV prevalence of females aged 20–24 is roughly three times greater than that of men in the same cohort[1]<br>▪ 10% of girls have acquired HIV by the time they leave school[3]<br><br>***Drivers:***<br>▪ Gender inequality, gender-based violence, sexual coercion, and gender discrimination<br>▪ Early sexual debut, often involving high rates of coerced sex<br>▪ Risky sexual behaviour (in part driven by the perception that HIV is now a treatable illness)<br>▪ Easy access to pornography through social media / internet<br>▪ Multiple partners and intergenerational/transactional relationships<br>▪ Limited condom use / challenges negotiating use<br>▪ The practice of dry sex<br>▪ Alcohol and substance abuse<br>▪ Poverty, poor nutrition, unemployment, and migration<br>▪ Inadequate access to quality education
Teenage pregnancy	***Context:***<br>▪ In 2016, 12% of adolescent girls (aged 15-19) had already given birth, with childbearing more common in rural areas than in urban areas (19% versus 14%)[4]<br>▪ There is higher unmet need for family planning in the 15–19 year old age group, compared with older women[4]<br>***Drivers:***<br>▪ Deeply entrenched social and cultural practices and concepts such as patriarchy expose adolescent girls to risk of early pregnancy<br>▪ Challenges with provision of comprehensive sex education in schools (often not taught or of poor quality)<br>▪ Insufficient awareness of risks / ignorance about safe sex practices<br>▪ Lack of understanding about the phenomenon of “blessers” and “sugar daddies”, and the extent to which this exposes adolescent girls and young women to increased risk of early pregnancy<br>▪ Lack of access to appropriate contraception and pregnancy termination<br>▪ Lack of access to and availability of youth-friendly services<br>▪ Low levels of protected sex (partly fuelled by alcohol and/or coercion)
School retention	***Context:***<br>▪ Low levels of education and high drop-out rates, especially in secondary school, 60% of learners do not graduate and most school dropout in South Africa occurs in grades 10

	and 11[5]<br>▪ For many South Africans living in poor rural and urban communities, teenage pregnancy is a particular risk factor, and accounts for 33% of drop-out among female learners[6]<br>***Drivers:***<br>▪ Overcrowded classrooms, poor teacher quality, lack of resources<br>▪ Dysfunctional family environments that fail to encourage school attendance / school performance<br>▪ A lack of interest in schooling, and previously failing a grade or being behind in school work<br>▪ Societal norms that expect adolescent girls and young women to take responsibility for their own welfare
Sexual and gender-based violence (SGBV)	***Context:***<br>▪ South Africa has among the highest level of SGBV in the world, with 17% of young women aged 18 to 24 experiencing violence from a partner in the 12 months before the survey[4]<br>▪ Violence in South Africa is estimated to cost the country over R28 billion (US$ 2 billion) per annum, amounting to 1% of GDP[7]<br>▪ An estimated 50 883 individuals, mostly women, experienced a sexual offence in 2016/17, and that the individuals who are at the highest risk of falling victim to sexual offences are women aged between 20 and 30[7]<br>***Drivers***<br>▪ Poverty and unemployment<br>▪ As a result of gender discrimination and their lower socio-economic status, adolescent girls and young women have fewer options and less resources at their disposal to avoid or escape abusive situations and to seek justice<br>▪ Cultural norms endorse patriarchal values<br>▪ Low status and power of girls and women<br>▪ Social norms related to masculinity which emphasise dominance and control over women (including through SGBV)<br>▪ Weak intersectoral coordination between key departments such as Justice, Health, Social Development, and the South African Police Service
Youth employment	***Context:***<br>▪ Youth unemployment in South Africa was 52% in the first quarter of 2018[8]<br>▪ 67% of unemployed women have been unemployed for a year or longer compared to 57% of their male counterparts[9]<br>▪ Persons who are not in employment, education or training (NEET) rate for 15-24 age group was 31% in 2016, rising to 32% in the first quarter of 2018[8,10]<br>***Drivers:***<br>▪ Weak economic growth in South Africa: real gross domestic product fell by 2% in the first quarter of 2018[20]<br>▪ Low labour absorption rates in South Africa's economy<br>▪ Poor alignment of systems such as the linkages between post-secondary school education and training systems, education and training authorities, and employers which makes it even difficult to tackle the skills mismatch problem

References

1. Avert. Global information and education on HIV and AIDS. https://www.avert.org/professionals/hiv-around-world/sub-saharan-africa/south-africa
2. Statistics South Africa. Mid-year population estimates. National Development Plan 2030, 2018. http://www.statssa.gov.za/publications/P0302/P03022018.pdf
3. Shisana O, Rehle T, Simbayi LC, et al. South African national HIV prevalence, incidence and behaviour Survey, 2012. HSRC Press, 2014.
4. The South Africa Demographic and Health Survey 2016, Key Indicators Report, National Department of Health.
5. Hartnack A. (2017) Background document and review of key South African and international literature on school dropout. DGMT.
6. Spaull N. (2015) Schooling in South Africa: How Low-quality Education Becomes a Poverty Trap. South African Child Gauge.
   Too costly to ignore – the impact of gender based violence in South Africa (2014) KPMG Human and Social Services https://assets.kpmg.com/content/dam/kpmg/za/pdf/2017/01/za-Too-costly-to-ignore.pdf
7. Optimus Study South Africa: Technical Report Sexual victimisation of children in South Africa Final report of the Optimus Foundation Study: South Africa May 2016.
8. Statistics South Africa, 2018, Labour Force Survey. Quarter 1 2018
9. IOL, https://www.iol.co.za/business-report/economy/52-of-south-africas-youth-has-no-job-14993687
10. Statistics South Africa, http://www.statssa.gov.za/?p=11129
